# Validated chromatographic approach for determination of two ternary mixtures in newly approved formulations for helicobacter pylori eradication: assessment of greenness profile and content uniformity

**DOI:** 10.1186/s13065-024-01215-1

**Published:** 2024-06-12

**Authors:** Yomna A. Salem, Samah A. Elsabour, Amal A. El-Masry

**Affiliations:** 1 Department of Pharmaceutical Chemistry, Faculty of Pharmacy, Sinai University, Kantara Branch, Ismailia, 41636 Egypt; 2 Department of Pharmaceutical Analytical Chemistry, Faculty of Pharmacy, Elsalehya El Gadida University, Elsalehya El Gadida, Sharkia, Egypt; 3 Department of Medicinal Chemistry, Faculty of Pharmacy, Mansoura University, Mansoura, 35516 Egypt

**Keywords:** Amoxicillin, Vonoprazan, Lansoprazole, Clarithromycin, Liquid chromatographic method, Tri-Pak, Capsule, Content uniformity, Eco-scale

## Abstract

A new, sensitive, and rapid isocratic reversed phase chromatographic method (RP-HPLC–UV) was developed for simultaneous separation of two newly co-formulated antiulcer mixtures; Amoxicillin, Vonoprazan and Clarithromycin [Mixture (I)], and Amoxicillin, Lansoprazole and Clarithromycin [Mixture (II)]. Analytical separation was performed using a Promosil C_18_ column and ultraviolet detection at 210 nm. The separation was achieved within only 8 min. For both mixtures, an aqueous solution, composed of (Acetonitrile: Methanol: 0. 2 M phosphoric acid) within ratio of (30: 30: 40) adjusted to final pH 3.0, was the mobile phase. This method was validated as per the International Conference on Harmonization guidelines. The linearity ranges of these proposed method of the (Mixture (I)) were 25.0–400.0 µg/mL Amoxicillin, 0.5–8.0 µg/mL Vonoprazan, and 12.5–200.0 µg/mL Clarithromycin. And the linearity ranges of the (Mixture (II)) were 10.0–300.0 µg/mL Amoxicillin, 0.3–9.0 µg/mL Lansoprazole and 5.0–150.0 µg/mL Clarithromycin. This method was firstly applied for effective separation of Amoxicillin, Vonoprazan and Clarithromycin [Mixture (I)]. It fulfilled good repeatability, sensitivity, and accuracy (R.S.D. < 2.0%). The mean recoveries of the analytes in their Tri-Pak formulations were acceptable. The greenness of the developed chromatographic methods was assessed using an Eco-scale method and it was applied for content uniformity testing as per the United States Pharmacopoeia (USP) and the acceptance value of Amoxicillin, in Mixture (I) was 2.88, the acceptance values for Amoxicillin, Lansoprazole in Mixture (II) were 2.592, 2.424, respectively.

**Supplementary Information:**

The online version contains supplementary material available at 10.1186/s13065-024-01215-1.

## Introduction

Helicobacter pylori {H. pylori} is one strain of bacteria which can invade the human stomach layer and neutralize the acidic medium of the stomach to be survived.

Recently, The Centers for Disease Control and Prevention monitoring that about two -third of world’s suffering from Helicobacter pylori {H. pylori} infection as this infection is popular in law countries [1, 2]. Chronic infection with H. pylori causes an atrophic gastritis (a kind of long lasting inflammation) which may cause certain type of stomach cancer and a rare type of non -Hodgkin lymphoma [3].

Two separate co-formulated Tri-Pak dosage forms of Amoxicillin (AMOX), Vonoprazan (VPZ), and Clarithromycin (CLA); [Mixture (I)], and AMOX, Lansoprazole (LAN), and CLA; [Mixture (II)] are used effectively to treat and prevent recurrent gastric and duodenal ulcers caused by certain types of bacteria (H. pylori). Many literature reviews confirmed the clinical superiority of these co-formulated ternary drugs over other marketed drugs in the effective eradication of recurrent helicobacter pylori with lower relapse incidence of duodenal ulcer preventing incidence of gastric cancer and lower susceptible infection to corona virus [4, 5].

Amoxicillin (AMOX, Fig. S1), (2S,5R,6R)-6-[[(2R)-2-amino-2-(4 hydroxyphenyl) acetyl] amino]-3,3-dimethyl-7-oxo-4-thia-1-azabicyclo [3.2.0] heptane-2-carboxylic acid, is a beta-lactam antibiotic [6]. It is used to treat bacterial infections [7], such as chest infections (including pneumonia) and dental abscesses [8]. It can also be used with other antibiotics to treat stomach ulcers [9]. Different reports were performed regarding the spectrophotometry [10–12], and spectrofluorimetry [13, 14]. Others, include high-pressure liquid chromatography (HPLC) [15, 16], electrochemical detection [17], ultra-pure liquid chromatography (UPLC) [18, 19], high pressure thin layer chromatography (HPTLC) [20], and gas chromatography (GC) [21].

Vonoprazan (VPZ) (Fig. S1), 1-[5-(2-Fluorophenyl)-1-(3-pyridinylsulfonyl)-1H-pyrrol-3-yl]-N-methylmethanamine, is a first-in-class potassium-competitive acid blocker medication, works by decreasing the amount of acid made in the stomach [6]. It is used mainly for the treatment of gastroduodenal ulcers (including some drug-induced peptic ulcers) and reflux esophagitis [8, 22, 23]. Few reports were performed for determination of VPZ using spectrophotometry [24, 25], spectrofluorimetry [26, 27], reversed phase high performance liquid chromatography (HPLC) [28, 29], capillary electrophoresis (CE), and electrochemical analysis [30], high performance liquid chromatography with tandem mass detection HPLC MS/MS [31].

Lansoprazole (LAN, Fig. S1), [(RS)-2-([3-methyl-4-(2,2,2-trifluoroethoxy)pyridin-2-yl]methylsulfinyl)-1H-benzo[d]imidazole], is a widely used proton pump inhibitor [6], which is as an active therapeutic agent in various formulations which reduces stomach acid. It is used to treat peptic ulcer disease, gastroesophageal reflux disease, and Zollinger–Ellison syndrome [8]. It is a racemic 1:1 mixture of the enantiomers dextro-lansoprazole and levo-lansoprazole. Dextro-lansoprazole is an enantiomerically pure active ingredient of a commercial drug. It has superior clinical efficacy in helicobacter eradication over levo-lansoprazole, which could has more advantage in preventing recurrent infection gastric cancer [32]. Several methods were carried out for separation of LAN enantiomers [33]. Different analytical separations were described for the determination of LAN either alone or in combination with other pharmaceuticals including spectrophotometry [12, 34, 35], spectrofluorimetry [36], electrochemical detection [37], potentiometry [38], reversed phase high performance liquid chromatography RP-HPLC [39–41], HPLC using mass detector [42, 43].

Clarithromycin (CLA), (3R,4S,5S,6R,7R,9R,11R,12R,13S,14R)-6-{[(2S,3R,4S,6R)-4-(Dimethylamino)-3-hydroxy-6-methyltetrahydro-2H-pyran-2-yl]oxy}[1]-14-ethyl-12,13-dihydroxy-4-{[(2R,4R,5S,6S)-5-hydroxy-4-methoxy-4,6-dimethyltetrahydro-2H-pyran-2-yl]oxy-7-methoxy-3,5,7,9,11,13 hexamethyloxacyclotetradecane-2,10-dion (Fig. S1), is an antibiotic drug which is used for the treatment of various bacterial infections [6], As skin infections, pneumonia, strep throat, H. pylori infection, and Lyme disease [8]. The literature shown that the analysis of CLA was performed through different techniques such as; spectrophotometry [44, 45], capillary electrophoresis [46], High performance thin layer chromatography (HPTLC) and HPLC [47, 48], liquid chromatography—mass spectrometry (LC/MS/MS) [49], ultra-performance liquid chromatography-mass spectrometry (UPLC MS/MS) [50, 51].

Up till now, there is no reported analytical method was developed for isocratic simultaneous determination of AMOX, VPZ, and CLA mixture and only one analytical method using HPLC method was attempted for the determination of AMOX and VPZ [52].

Only two reported HPLC methods were published for the determination of AMOX, LAN, and CLA (Mixture (II)), both methods are time consuming and with sophisticated condition for separation [53, 54].

## Aim of work

This paper aimed to develop newly liquid chromatographic method to separate effectively and quantitate two ternary drug mixtures in their raw materials and Tri-Pak dosage forms which could be further applied in quality control labs to ensure the validity of pharmaceutical marketing products and enhance clinical outcomes. For the second mixture, our developed method has many advantages over the published one that the separation is established by applying one measured wavelength in a shorter run time which is not exceed 8 min, unlike the published reference method (19 min) with multiple wavelengths. Besides, it is an environmentally green as it requires lower amount of the organic modifier intake compared to the second published method. By comparing the Eco- score of this method with the other published methods to evaluate the greenness of the analytical method, it was found that the proposed method is greener in terms of usage of hazardous solvents, energy consumption, and production of waste. This paper developed a well validated, sensitive and time saving chromatographic method to separate and quantitate two ternary mixture drugs which could be extended to good applicability of good content uniformity, according to USP guidelines in their two separate tri-Pak formulations.

## Experimental

### Materials and methods

#### Chemicals and reagents

- AMOX (99.97%), LAN (99.80%) and CLA (99.5%) were provided by EIPICO pharmaceutical industries company, Egypt. They were utilized directly without further purification.
- VPZ (99.2%) was provided by Zeta pharmaceutical industries company, Egypt.
- Voquezna Tri-Pak, (label claim: 1000.0 mg AMOX (2*500.0 mg individual capsules), 20.0 mg VPZ per tablet, 500.0 mg of CLA per tablet) manufactured by Phathom, was obtained from commercial source.
- Prevac Tri-Pak, (label claim: 1000.0 mg AMOX (2* 500.0 mg individual capsules), 30.0 mg LAN per capsule, 500.0 mg of CLA per tablet) manufactured by Citron, was obtained from commercial source.
- Both Triethyl amine (TEA) and Orthophosphoric acid (OPA) were obtained from Prolabo (Paris, France).
- Acetonitrile (ACN) and Methanol (MeOH) (HPLC grade) were obtained from Sigma-Aldrich Chemie GmbH (Steinheim, Germany).
- Deionized water was obtained using Millipore highly purified system.

### Instrumentation

Perkin Elmer TM “Series 200” High performance Chromatograph was used for the separation, it was equipped with a UV/VIS detector, and the selected wave length for detection was 210 nm. The software used for data processing was Total Chrom Workstation (Massachusetts, USA). Millipore filter (Sibata) was used for filtration of the mobile phase. Consort NV P-901 pH –Meter (Belgium) was used for pH measurements.

### Chromatographic conditions

The optimum chromatographic condition for separation of both mixtures was obtained by using Promosil C_18_ column (250 mm × 4.6 mm. 5 μm) and mobile phase ACN: MeOH: 0.2 M OPA (30: 30: 40) pumped with a flow rate of 1.0 mL/min, the detection wavelength was 210 nm for {Mixture [I] and Mixture (II)}. Mixing of the mobile phase was carried out using an ultrasonic bath for thirty minutes. The mobile phase was than filtered by using 0.45 µm membrane filter.

### Stock standard solution (mixture [I and II)]

Stock standard solution of each drug was prepared by weighting and dissolving each powder of two ternary mixture drugs separately in 100 mL measuring flask, for AMOX 200.0 mg was dissolved in ultra-deionized water to produce 2000.0 μg/mL solution, 20.0 mg of VPZ and 100.0 mg of CLA were dissolved in methanol to produce 200.0 μg/mL and 1000.0 μg/mL solutions, respectively and 30.0 mg of LAN was dissolved in acetonitrile to produce 300.0 μg/mL solution.

These prepared stock solutions were further diluted using the same HPLC grade solvents to prepare throughout the study; working solutions of 1000.0 μg/mL of AMOX, 100.0 μg/mL, 150.0 μg/mL, 500.0 μg/mL of VPZ, LAN and CLA, respectively. These solutions were stored at 3  C and it was protected from direct exposure to light.

### Procedures

#### Preparation of calibration curves (Mixture [I and II])

To study the linearity, the concentration ranges of 25.0–400, 0.5–8.0 and 12.5–200.0 μg/mL of AMOX, VPZ, and CLA, respectively for Mixture (I) and 10.0–300.0, 0.3–9.0 and 5.0–150.0 μg/mL of AMOX, LAN and CLA, respectively for Mixture (II) were prepared by transferring specific aliquots from their working solutions separately into 10 mL volumetric flasks. The volumes were completed using a mobile phase, the solutions were filtered. Then, twenty microliters were injected into HPLC. Peak area of each drug was plotted against each drug final concentration. The regression equation of each line was calculated.

#### Determination of the AMOX, VPZ, and CLA [Mixture (I)] and AMOX, LAN, and CLA [Mixture (II)] in their synthetic mixtures

To prepare synthetic mixtures, transfer aliquots of AMOX, VPZ, and CLA and AMOX, LAN, and CLA from their working solutions into specified series of 10 mL volumetric flasks, using the same ratio as per the pharmaceutical formulation; 10.0: 0.2: 5.0 [Mixture (I)] and 10.0:0.3: 5.0 [Mixture (II)] and the procedures under “Preparation of calibration curves” were performed. The percentage of recoveries were mathematically calculated from the corresponding regression equation or derived from the developed calibration graphs.

#### Application to combined dosage forms

Ten individual capsules and tablets given from each separated co-formulated Tri-Pak dosage form were weighed and pulverized well. An amount of powder equivalent to 1000.0 mg AMOX, 20.0 mg VPZ, 500.0 mg of CLA, and 1000.0 mg AMOX, 30.0 mg LAN and 500.0 mg of CLA (as the same ratio of the drugs in Tri-Pak dosage form) for mixtures (I) and (II) were transferred into two separate 100 mL volumetric flasks. 80 mL of methanol was added and the flasks were sonicated for 20 min, then the flasks were completed to volume using mobile phase. 1 ml of each solution was added to 10 mL volumetric flask and the volume was completed with mobile phase.

Filtration of all the prepared samples was double-carried out through 0.45 μm filters and the samples were injected into the HPLC chromatographic system.

Evaluation of the various concentrations within the developed calibration range throughout the whole study was carried out by picking three samples for each drug and the all steps under “Construction of calibration curves” were performed. Furthermore, the nominal content of their combined Tri-Pak dosage forms was calculated using corresponding regression equation.

## Results

### Chromatographic conditions

Different columns and mobile phases were examined to separate the studied mixtures with good chromatographic system suitability parameters. Promosil C_18_ column (250 mm × 4.6 mm. 5 μm) at temperature of 25 °C was selected as the most suitable column condition for separation of the combined triple dosage forms of different mixtures. The mobile phase composed of ACN: MeOH: 0.2 M ortho-phosphoric acid OPA (30: 30: 40) at pH 3.0 pumped with a flow rate 1.0 mL/min was found to be the most suitable mobile phase for good separation of the studied mixtures. The UV detection wave length of the both mixtures was 210 nm.

**Fig. 1:**
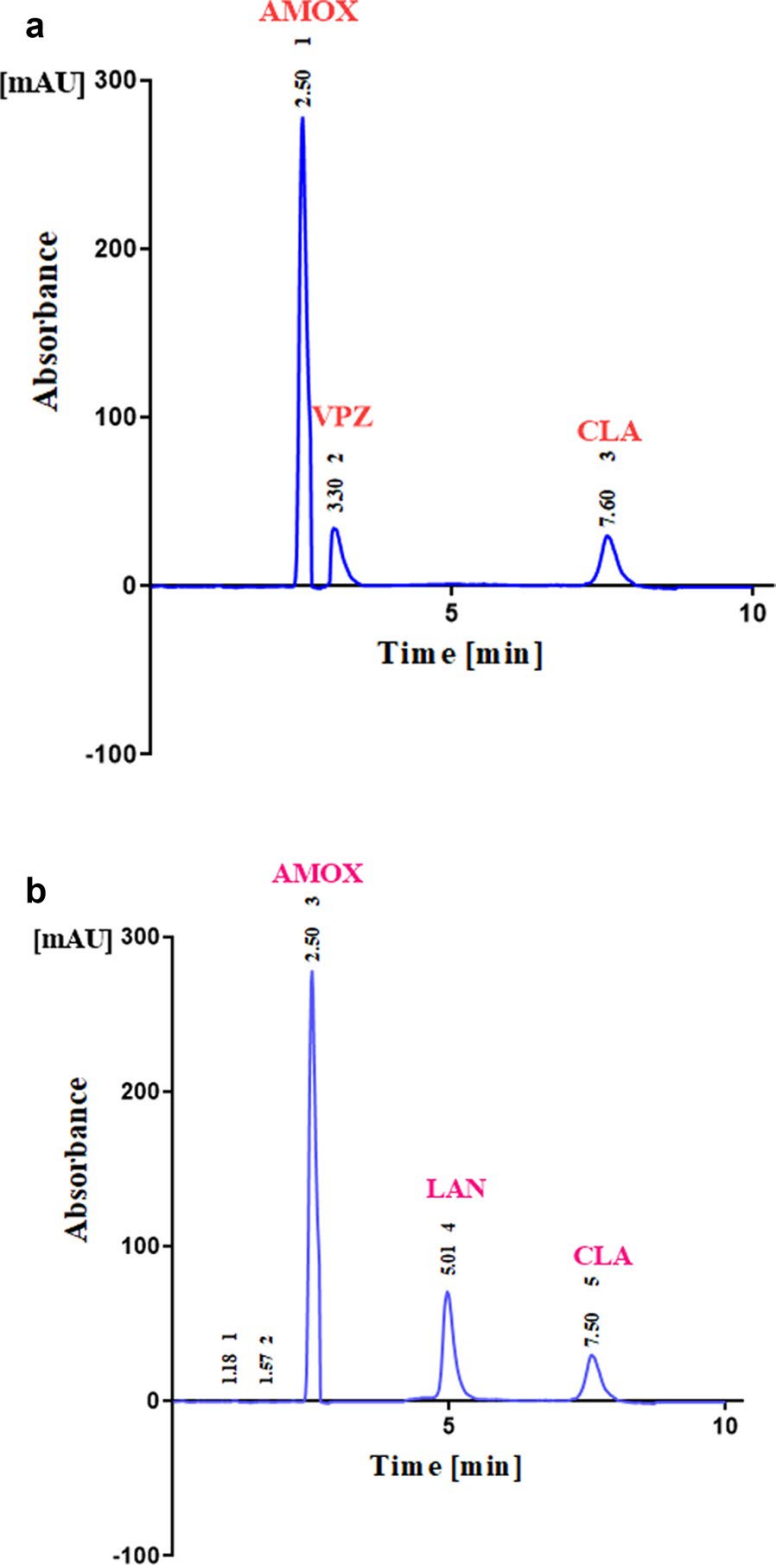
**a** Typical chromatogram for the separation of AMOX (200.0 µg/mL, 2.5 min), VPZ (4.0 µg/mL, 3.3 min), and CLA (100.0 µg/mL, 7.6 min), in synthetic mixture (I). Chromatographic system: Promosil C_18_ column (250 mm × 4.6 mm. 5 μm). Mobile phase mobile phase consisting of ACN: MeOH: 0.2 M OPA (30: 30: 40) at pH 3.0. Flow rate; 1.0 mL/min, UV detection at 210 nm. **b** Typical chromatogram for the separation of AMOX (200.0 µg/mL, 2.5 min), LAN (6.0 µg/mL, 5.01 min), and CLA (100.0 µg/mL, 7.5 min), in synthetic mixture (II). Chromatographic system: Promosil C_18_ column (250 mm × 4.6 mm. 5 μm). Mobile phase mobile phase consisting of ACN: MeOH: 0.2 M OPA (30: 30: 40) at pH 3.0. Flow rate; 1.0 mL/min, UV detection at 210 nm

**Fig. 2:**
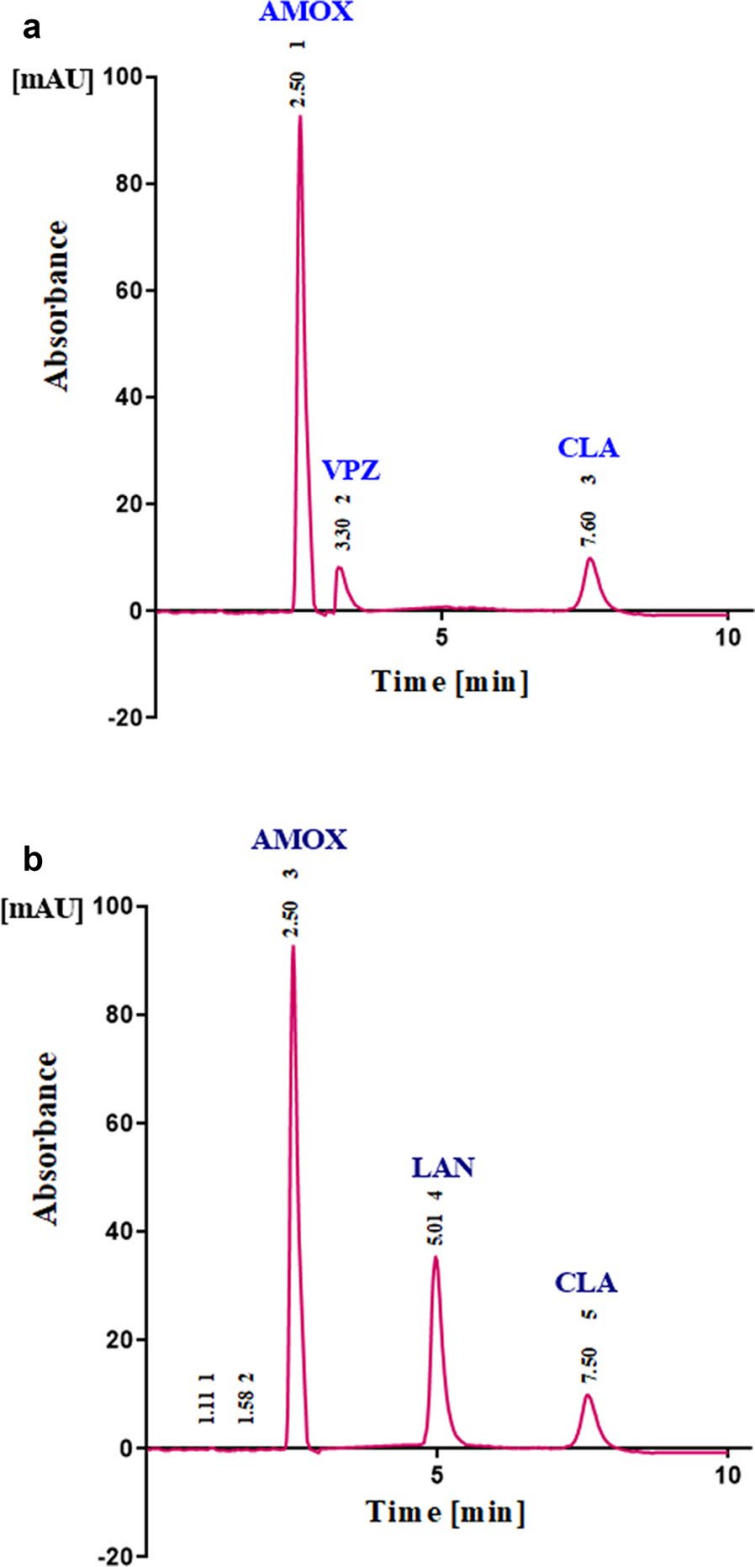
**a** Typical chromatogram for the separation of AMOX (100 µg/mL, 2.5 min), VPZ (2.0 µg/mL, 3.3 min), and CLA (50.0 µg/mL, 7.6 min), in combined Tri-Pak dosage form. Chromatographic system: Promosil C_18_ column (250 mm × 4.6 mm. 5 μm). Mobile phase mobile phase consisting of ACN: MeOH: 0.2 M OPA (30: 30: 40) at pH 3.0. Flow rate; 1.0 mL/min, UV detection at 210 nm. **b** Typical chromatogram for the separation of AMOX (100.0 µg/mL, 2.5 min), LAN (3.0 µg/mL, 5.01 min), and CLA (50.0 µg/mL, 7.5 min), in combined Tri-Pak dosage form. Chromatographic system: Promosil C_18_ column (250 mm × 4.6 mm. 5 μm). Mobile phase consisting of ACN: MeOH: 0.2 M OPA (30: 30: 40) at pH 3.0. Flow rate; 1.0 mL/min, UV detection at 210 nm

The suggested LC method provides a good separation between AMOX, VPZ, and CLA [Mixture (I)], and AMOX, LAN, and CLA [Mixture (II)] with acceptable chromatographic system suitability parameters in a reasonable elution time. The separation chromatogram of these studied drugs are shown in Figs. 1a, b and 2a, b for Mixture (I) and mixture (II) in their synthetic mixtures and combined Tri-Pak formulations, respectively. The chromatographic system suitability parameters represented in (Table s1a, b).

## Discussion

As these mixtures have high overlapped UV spectra, Fig S2, the HPLC is the best choice for their separation. The resolution efficiency of HPLC was utilized for the separation of AMOX, VPZ and CLA (Mixture (I)), and AMOX, LAN and CLA (Mixture (II)) which was confirmed by analyzing the synthetic prepared mixtures. Several trials were carried out to obtain a good resolution between these ingredients. These trials included using different ratios of the mobile phase, trying different flow rates for pumping the mobile phase and using different wavelengths for detection until the chromatographic conditions were optimized (Table S1a, b).

### Method development

Applying USP guidelines [55] for effective measurement of chromatographic performance. The chromatographic parameters most affected the separation of these two mixtures selected carefully and measured as presented in Table S1a, b.

### The stationary phase

The choice of the optimum column is a critical element in developing effective separation of the two ternary mixtures with high resolution and comparable run time. Two columns were assessed for mixture separation, including: CN column (150 mm × 4.6 mm, 3 μm) and Promosil C_18_ column (250 mm × 4.6 mm, 5 μm). Well-resolved peaks with an acceptable symmetry and effectively were achieved within short elution time using Promosil C_18_ column. In addition, the CN column was incapable of affording well-separated bands in a reasonable time for the studied drugs.

### Mobile phase

#### The pH effects

Increasing pH over the range; 3.0 to 6.0 range was done by using an increasing amount of diluted triethylamine solution. The ionization and hydrophobicity constants of the studied drugs in mixtures were presented by their pKa and log P (octanol/ water) values, respectively. AMOX has a log P value of − 2.3 and two pKa values of 3.23 and 7.22 [57], VPZ has a log P value of 2.03 and pKa value of 9.01 [57], LAN has a log P value of 3.03 and pKa value of 9.35 and7.16 [57], while CLA has a log P value of 3.24 and two pKa values of 9 and 12.46 [57]. A minimal effect on the elution times of AMOX, VPZ, LAN, and CLA was observed by increasing the pH value of both mixtures to 6.0, as these basic drugs will be ionized over the studied pH range. Further increase in pH to 6.0, disturb the symmetry of the peaks of studied drugs with increased runtime. As represented at Table S 1a, b, the optimum pH for separation of both mixtures was pH 3.0. This pH affords acceptable resolution in a reasonable run time (7.9 min) with maximum peak efficiency (Fig. S3).

#### Organic modifier type and ratio

Various organic modifiers were tested for good separation; as ethanol, methanol, acetonitrile and n-propanol. Methanol and Acetonitrile were preferred over the other modifiers as they provide well-separated symmetrical peaks within reasonable run time. Meanwhile, using methanol and acetonitrile alone were increase the analysis times up to 20 min and cause a distraction of peak shape. As a result of the several experimental trials, a mixture of both solvents ACN, and MeOH was selected. Various ACN: MeOH concentrations were increased from 25 to 35%. (Table S1a, b). The percentage ratio 30: 30% (v/v) was selected as the optimum ratio as it afforded the ideal separation of the studied drugs within run time of eight minutes, accompanied with higher sensitivity and maximum efficiency as revealed by higher numerical value of theoretical plates as shown in Figs. S4, S5.

#### The choice of detection wavelength

As given in table S 1 a, b**,** the chromatographic peaks of the studied mixtures were tested over various wavelengths as 210, 220, 230, 281 nm (Fig S2). The optimum wavelength for the UV detection for two ternary mixture drugs was best adjusted to 210 nm for [Mixture (I)] and [Mixture (II)]. The higher detection limit of the studied drugs at this wavelength permits the easily determination of the mixture at their medicinally recommended ratio without interference from excipients in their dosage forms (Fig. S6).

#### Flow rate

Different ranges of flow rates from 0.8 to 1.2 mL/min (Table S1a, b) were investigated to determine their impact on resolving of the provided mixtures, a flow rate of 1.0 mL/min was the best selected for the separation of the two studied mixture drugs in short elution time accompanied with higher efficiency as presented in Fig. S7.

#### Method validation

These methods were validated according to the International Conference on Harmonization (ICH) guidelines [56].

### Linearity and range

The linear relation between the peak area of AMOX, VPZ and CLA (Mixture (I)), and AMOX, LAN and CLA (Mixture (II)) and the concentration ranges of 25.0–400.0 µg/mL, 0.5–8.0 µg/mL and 12.5–200.0 µg/mL for AMOX, VPZ, and CLA (Mixture (I)) and ranges of 10.0- 300.0 µg/mL, 3–9.0 µg/mL and 5.0–150.0 µg/mL for AMOX, LAN, and CLA [Mixture (II)], respectively were studied, Tables 1, 2.

**Table 1:** Performance data for the determination of the studied mixture (I) by the proposed aqueous liquid chromatographic method

Parameter	AMOX	VPZ epimer	CLA
Concentration range (μg/mL)	25.0–400.0	0.5–8.0	12.5–200.0
Correlation coefficient	0.9999	0.9999	0.9999
LOD (μg/mL)	1.41	0.058	1.556
LOQ (μg/mL)	4.271	0.175	4.715
% RSD	0.522	0.607	0.537
% Error	0.213	0.247	0.219

N.B. % Error = RSD%/√ n

**Table 2:** Performance data for the determination of the studied mixture (II) by the proposed aqueous liquid chromatographic method

Parameter	AMOX	LAN	CLA
Concentration range (μg/mL)	10.0–300.0	0.3–9.0	5.0–150.0
Correlation coefficient	0.9999	0.9999	0.9999
LOD (μg/mL)	1.545	0.026	0.433
LOQ (μg/mL)	5.099	0.079	1.312
% RSD	0.496	0.341	0.538
% Error	0.202	0.139	0.220

N.B. % Error = RSD%/√ n

The regression equations and all data analysis of regression line for the drugs in Mixture (I) were represented in (Table 1) as following:

1 $${\text{PA}}\,{ = }\,{15}.{173} + {1}.{\text{684C}}\,\,\left( {{\text{r}}\,{ = }\,{0:9999}} \right)\,\,{\text{for AMOX}}$$

2 $${\text{PA}}\,{ = }\,{19}{\text{.65}} + 6.1{\text{C}}\,\,\left( {{\text{r}}\,{ = }\,{0:9999}} \right)\,\,{\text{for VPZ}}$$

3 $${\text{PA }} = {4}.{423 } + \, 0.{7}0{\text{6C }}\left( {{\text{r }} = 0:{9999}} \right){\text{ for CLA}}$$

The regression equations and all data analysis of regression line for the drugs in Mixture (II) were represented in (Table 2) as following:

4 $${\text{PA }} = { 21}.{51 } + { 1}.{\text{663 C }}\left( {{\text{r }} = 0:{9999}} \right){\text{ for AMOX}}$$

5 $${\text{PA }} = - {4}.{8}\, \times \,10^{ - 4} + 5.528{\text{C}}\,\left( {{\text{r }} = 0:{9999}} \right)\,\,{\text{for LAN}}$$

6 $${\text{PA }} = { 5}.{362 } + \, 0.{\text{693 C }}\left( {{\text{r }} = 0:{9999}} \right){\text{ for CLA}}$$

where PA is the Peak area, C is the drug concentration (μg/mL), and r is the regression coefficient.

### Limit of detection (LOD) and limit of quantification (LOQ)

AS per ICH Q2 (R1) guidelines [56], the following equation was used to calculate LOD and LOQ.

7 $${\text{LOD }} = { 3}.{3}\,\,{{{\text{Sa}}} \mathord{\left/ {\vphantom {{{\text{Sa}}} {\text{b}}}} \right. \kern-0pt} {\text{b}}}$$

8 $${\text{LOD }} = { 10}\,\,{{{\text{Sa}}} \mathord{\left/ {\vphantom {{{\text{Sa}}} {\text{b}}}} \right. \kern-0pt} {\text{b}}}$$

where Sa = the standard deviation of the response, b = the slope of calibration curve. LOD and LOQ values for AMOX, VPZ, and CLA [Mixture (I)], and AMOX, LAN, and CLA [Mixture (II)] were calculated using the above equations and the results were given in Tables 1, 2.

### Accuracy and precision

To investigate the accuracy and precision of the developed chromatographic methods, three determinations for three concentrations of AMOX, VPZ, and CLA [Mixture (I)] and AMOX, LAN, and CLA [Mixture (II)] at three successive days were considered as an inter-day precision. And triplicated assessment for three different concentrations within the same day is considered as an intraday precision, results are presented in Table 3 a, b. The lower values of SD and RSD proved the high precision of LC method. Furthermore, small values of ‘% Error” exhibit excellent accuracy.

**Table 3:** Accuracy and precision data for the determination of the studied mixtures by the proposed aqueous liquid chromatographic method

(a)		AMOX concentration (μg/mL)			VPZ Concentration (μg/mL)			CLA Concentration (μg/mL)		
		100.0	200.0	300.0	2.0	4.0	6.0	50.0	100.0	150.0
Intra-day	x̄^a^	100.27	99.79	99.69	99.48	100.04	100.87	99.84	99.20	100.76
	± SD	0.55	0.29	0.37	0.49	0.26	0.87	0.52	0.20	0.84
	% RSD	0.55	0.29	0.37	0.49	0.26	0.86	0.52	0.21	0.83
	% Error	0.32	0.17	0.21	0.28	0.15	0.50	0.30	0.12	0.48
Inter-day	x̄^a^	100.19	99.67	99.82	99.51	100.02	100.75	99.93	99.23	100.82
	± SD	0.37	0.54	0.73	0.49	0.17	0.27	0.58	0.71	0.18
	% RSD	0.37	0.54	0.73	0.49	0.17	0.27	0.58	0.72	0.18
	% Error	0.21	0.31	0.42	0.29	0.1	0.15	0.34	0.42	0.11

(b)		AMOX Concentration (μg/mL)			LAN concentration (μg/mL)			CLA concentration (μg/mL)		
		60.0	120.0	180.0	1.8	3.6	5.4	30.0	60.0	90.0
Intra-day	x̄^a^	99.93	99.87	99.72	99.53	99.71	100.55	100.08	100.58	99.18
	± SD	0.33	0.20	0.44	0.64	0.66	0.79	0.91	0.62	0.93
	% RSD	0.33	0.20	0.44	0.65	0.66	0.78	0.91	0.61	0.94
	% Error	0.19	0.12	0.26	0.37	0.38	0.45	0.52	0.35	0.54
Inter-day	x̄^a^	99.96	99.85	99.75	99.64	99.72	100.56	100.06	100.50	99.19
	± SD	0.34	0.58	0.62	0.95	1.07	0.89	0.64	0.63	0.78
	% RSD	0.34	0.58	0.62	0.96	1.08	0.88	0.64	0.63	0.78
	% Error	0.20	0.34	0.36	0.55	0.62	0.51	0.37	0.36	0.45

^a^ Each result is the mean recovery of three separate determinations

SD = Standard deviation

RSD = Relative standard deviation

For the synthetic mixtures and novel Tri-Pak dosage forms, the results of analysis of AMOX, VPZ, and CLA and AMOX, LAN, and CLA in their separate mixtures using the suggested chromatographic method were compared with HPLC comparison method for mixtures I [52] and reference method [53] for Mixture (II), respectively. The comparison is performed using both variance ratio F-test and the student's t-test [58]. In terms of precision and accuracy, the data shown in Tables 4, 5a, b successfully revealed no significant difference between the performance validity of the proposed and the comparison methods.

**Table 4:** Assay results for the determination of the studied synthetic mixtures by the proposed aqueous liquid chromatographic method

(a)	Proposed HPLC method						Reference method [52]		
	Conc. taken (μg/mL)			% Found^a^			% Found*		
	AMO	VPZ	CLA	AMO	VPZ	CLA	AMO	VPZ	CLA
Data	100.0	2.0	50.0	99.66	99.08	99.92	101.7	100.1	102.6
	200.0	4.0	100.0	99.79	99.48	99.23	100.5	102.3	98.3
	300.0	6.0	150.0	100.27	100.04	100.89	99.7	101.4	97.4
${\overline{\text{X}}}$				99.91	99.53	100.01	100.63	101.27	99.43
± SD				0.32	0.48	0.83	1.01	1.11	2.78
t- value				1.19 (2.78)	2.49 (2.78)	0.35 (2.87)			
*F-*value				9.8 (19)	5.26 (19)	11.11 (19)			

(b)	Proposed HPLC method						Reference method [53]		
	Conc. taken (μg/mL)			% Found^a^			% Found*		
	AMO	LAN	CLA	AMO	LAN	CLA	AMO	LAN	CLA
Data	100.0	3.0	50.0	99.98	99.78	100.01	102.5	99.42	98.6
	200.0	6.0	100.0	99.93	99.58	99.62	99.6	99.76	96.3
	300.0	9.0	150.0	100.95	99.65	100.54	99.5	99.15	95.4
${\overline{\text{X}}}$				100.29	99.67	100.06	100.53	100.56	96.77
± SD				0.58	0.10	0.46	1.70	1.69	1.65
t- value				0.24 (2.78)	1.22 (2.78)	3.33 (2.78)			
*F-*value				8.78 (19)	9.07 (19)	12.77 (19)			

^a^ Each result is the average of three separate determinations

**Table 5:** Assay results for the determination of the studied mixtures in their combined Tri-Pak dosage forms by the proposed aqueous liquid chromatographic method

(a)		Proposed HPLC method						Reference method [52]		
		Conc. taken (μg/mL)			% Found^a^			% Found^a^		
Voquenza Tri-Pak		AMO	VPZ	CLA	AMO	VPZ	CLA	AMO	VPZ	CLA
	Data	75.0	1.5	37.5	99.76	101.07	99.76	102.6	98.4	99.63
		150.0	3.0	75.0	100.5	99.65	98.95	101.4	97.9	98.74
		225.0	4.5	112.5	98.43	100.34	100.88	100.9	101.02	100.42
	${\overline{\text{X}}}$				99.56	100.35	99.86	101.63	99.11	99.60
	± SD				1.05	0.71	0.97	0.87	1.68	0.84
	t- value				2.63 (2.78)	1.17 (2.78)	0.36 (2.78)			
	*F-*value				1.44 (19)	5.75 (19)	1.33 (19)			

(b)		Proposed HPLC method						Reference method [53]		
		Conc. taken (μg/mL)			% Found^a^			% Found^a^		
Prevac Tri-Pak		AMO	LAN	CL	AMO	LAN	CLA	AMO	LAN	CLA
	Data	75.0	2.25	37.5	101.08	100.91	101.07	102.3	98.8	100.8
		150.0	4.5	75.0	99.61	100.03	100.65	99.8	99.9	99.7
		225.0	6.75	112.5	99.08	98.76	100.88	100.1	102.2	100.44
	${\overline{\text{X}}}$				99.92	99.90	100.87	100.73	100.30	100.31
	± SD				1.04	1.08	0.21	1.37	1.74	0.56
	t- value				0.82 (2.78)	0.34 (2.78)	1.6 (2.78)			
	*F-*value				1.74 (19)	2.58 (19)	7.11 (19)			

^a^Each result is the average of three separate determinations

Robustness was tested by applying the proposed methods with the intentional minor modifications in the chromatographic conditions and measure the steadiness of the peak area values “Remaining of the of peak areas values unchanged or only slightly changed by a slight variation of the separation conditions”. This study includes pH (3.0 ± 0.1), ACN proportion (30 ± 1% v/v), MeOH proportion (30 ± 1% v/v) and OPA concentration (0.2 ± 0.02 M). These intentional minor modifications did not disturb the peak area values of all drugs in the two mixtures Table S2.

### Selectivity and specificity

To ensure selectivity of the method, the interference of the additives in the two dosage combinations with the studied drugs were verified by applying the proposed LC method to placebo. The placebo peak had zero reading at 210 nm revealing the selectivity of the method that there was no intrusion from the combined Tri-Pak dosage form excipients**.**

### System suitability

As per USP guideline [55], the system suitability parameter calculations could be efficiently measured and performed in term of column performance which is measured as (number of theoretical plates, N) and should be > 2000 & resolution factor (Rs) which should be > 1.5. During the process of developing our chromatographic method, System suitability variables examination are critical additive to ensure the effective performance of operational developed system (Table 1Sa, b).

#### Applications

### Statistical analysis

AMOX, VPZ, and CLA and AMOX, LAN, and CLA were determined in their synthetic mixtures by the developed novel LC and the results are represented (Tables 4, 5a). The given data were statistically analyzed and the accuracy and precision were confirmed by the high recovery percent values which assure the applicability of the developed methods for the quality control analysis of two ternary mixture drugs either separately or in any of their combined formulations (Fig. 2a, b).

### Analysis of AMOX, VPZ, and CLA and AMOX, LAN, and CLA in their combined Tri-Pak formulations

Determination of AMOX, VPZ, and CLA and AMOX, LAN, and CLA in their novel Tri-Pak dosage forms “in their clinical doses” of 2. 10.0: 0.2: 5.0 and 10.0:0.3: 5.0 for Mixture (I) and (II), respectively, were assessed by the proposed LC methods. The measured data presented in Tables 4, 5b were assuring the validity of the proposed methods in routine quality control analysis of these drugs in their mixtures (Fig. 2a, b).

### Application of content uniformity test

One of the advantages of this developed method is a short consuming run time which is not exceed eight minutes, so the content uniformity testing was ideally applied to estimate the concentration of AMOX in Mixture (I) and AMOX and LAN in Mixture (II) in their capsules, packed separately in Tri-Pak formulations. The test of content uniformity as recommended by of USP protocol were carried out and the results are shown in Table S3. The acceptance value (AV) for the pharmaceutical packed capsule was calculated and found to have lower numerical values than the acceptance value (LI), and the acceptance values were 2.88. For AMOX, in Mixture (I) and 2.592, 2.424 for AMOX, LAN in Mixture (II), which assures the content uniformity, and the results were extended to further application in quality control labs.

The previously published methods [52–54] could not be used for content uniformity measurement for this encapsulated drug in their Tri-Pak dosage forms.

### Assessing the greenness of the proposed method: the analytical eco-scale

Evaluation and agreement of the method with green chemistry was assessed according to Eco- scale tool which is concerned with type and volume of the solvent, haziness of the solvent, instruments energy consumption waste. To evaluate the method according to Eco- scale, each point of these items is given penalty points, then the summation of penalty points is subtracted from 100 (stands for the ideal green method) [59, 60]. The higher the score (near 100), the greener the method is. The score of the proposed method is 81 which is an indication of the good greenness of the proposed method. Comparing the Eco- scale of the proposed method with the published method [53], it was found that the proposed method has the higher Eco- scale. Thus, it could be used for routine analysis of the studied mixture without harming the environment (Table S4).

## Conclusion

The newly developed chromatographic methods of the novel studied mixtures used for helicobacter eradication in recurrent severe ulcers accompanied by gastric cancer and corona infections were completely validated and could be effectively applied for the routine analysis in quality control laboratories where time-saving and well-evaluated fulfillment of the whole chromatographic performance were achieved throughout the development and evaluation of the method. The suggested RP-HPLC is the first chromatographic method for the determination of Mixture (I) with higher quantitation limits and excellent resolution. Also, it enhances the clinical outcomes and ensures the quality and validity of pharmaceutical separate tri-pack formulations in the pharmaceutical market. Furthermore, they rapidly separate and quantify two ternary mixture drugs in their challenging ratios of pharmaceutical formulation with higher sensitivity and good acceptance values to fulfill content uniformity, according to USP guideline. It has excellent Eco- scale and can applied to routine analysis of the mixtures without harming the environment.

### Supplementary Information

Supplementary material file1.

## Data Availability

Data generated or analyzed during this study are available from the corresponding author upon reasonable request.

## Abbreviations

LC: Liquid chromatographic method
AMOX: Amoxicillin
VPZ: Vonoprazan
LAN: Lansoprazole
CLA: Clarithromycin
ICH: International council for harmonization
% RSD: Percentage relative standard deviation
LOD: Limit of detection
LOQ: Limit of quantification
