# Supplementary material for: Validated chromatographic approach for determination of two ternary mixtures in newly approved formulations for helicobacter pylori eradication: assessment of greenness profile and content uniformity
Source: BMC Chem. 2024 Jun 12;18(1):111. doi: 10.1186/s13065-024-01215-1 (PMC11167897; doi:10.1186/s13065-024-01215-1)
Supplement: Supplementary file 1 — Supplementary material file1. [file 13065_2024_1215_MOESM1_ESM.docx]

# Validated chromatographic approach for determination of two ternary mixtures in newly approved formulations for Helicobacter pylori eradication: Assessment of greenness profile and content uniformity.

**Yomna A. Salem^1*^, Samah** **A. Elsabour^2^ and Amal A. El-Masry^3*^**

1. Yomna A. Salem (***Corresponding author**)

Lecturer, Department of Pharmaceutical Chemistry, Faculty of Pharmacy, Sinai University − Kantara Branch, Ismailia 41636, Egypt.

*Email:* [*yomnasalem26@gmail.com*](mailto:yomnasalem26@gmail.com) *&* [*Yomna.abdulaziz@su.edu.eg*](file:///C:\Users\Laptop%20Shop\Downloads\Yomna.abdulaziz@su.edu.eg)

*Tel. +201023622361*

1. Samah A. Elsabour

Associate Professor, Department of Pharmaceutical Analytical Chemistry, Faculty of Pharmacy, Elsalehya El Gadida University, Elsalehya El Gadida, Sharkia, Egypt.

*Email:* [*samahsabour@gmail.com*](mailto:samahsabour@gmail.com)

1. Amal A. El-Masry (***Corresponding author**)

Lecturer, Department of Medicinal Chemistry, Faculty of Pharmacy, Mansoura University, Mansoura, 35516, Egypt.

*Email*: [dr.Amal90@mans.edu.eg](mailto:dr.Amal90@mans.edu.eg)

**Amoxicillin (AMOX) Lansoprazole (LAN)**

**Vonoprazan (VPZ) Clarithromycin (CLA)**

**Fig. S1.** Structural formula of Amoxicillin (AMOX), Lansoprazole (LAN), Vonoprazan (VPZ), and Clarithromycin (CLA).


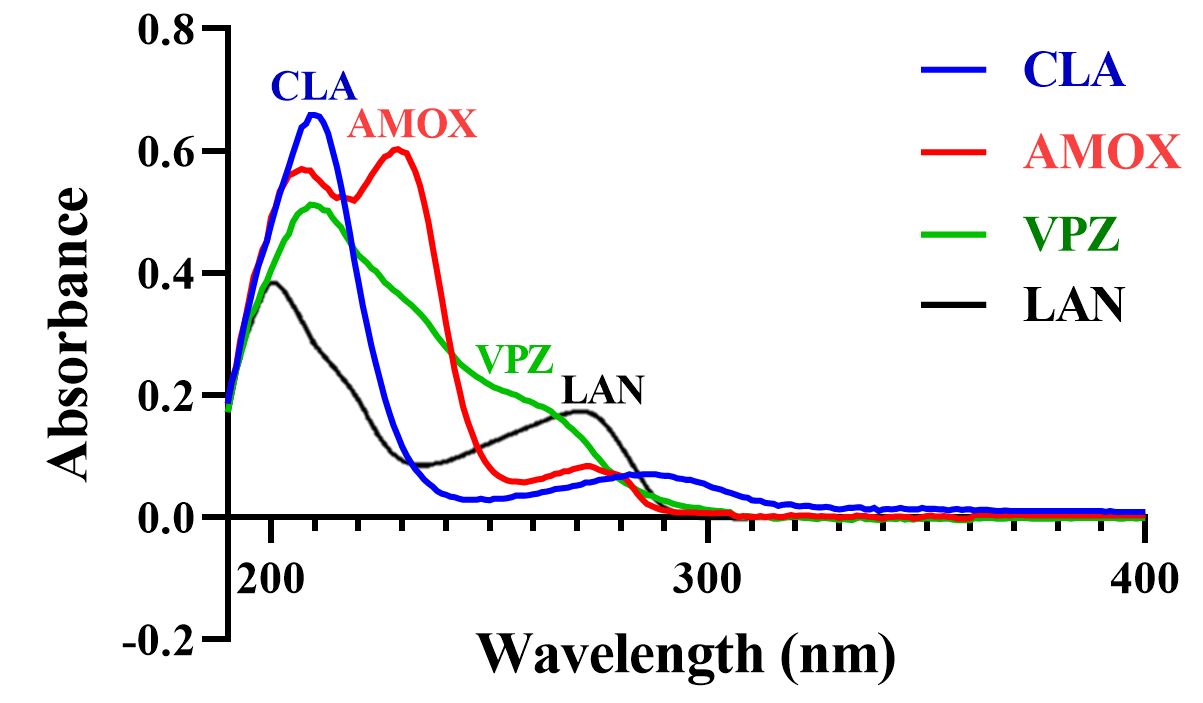


**Fig. S2.** Uv-Vis spectra of Amoxicillin (AMOX), Lansoprazole (LAN), Vonoprazan (VPZ), and Clarithromycin (CLA)

**Fig. S3.** Effect of different pH on the number of theoretical plates of AMOX (400.0 µg/mL), VPZ (10.0 µg/mL), LAN (15.0 µg/mL) and CLA (250.0 µg/mL) using mobile phase consisting of ACN: MeOH: 0.2 M OPA (30: 30: 40). Flow rate, 1.0 mL/min.

**Fig. S4.** Effect of different % concentration of ACN on the number of theoretical plates of AMOX )400.0 µg/mL(, VPZ )10.0 µg/mL(, LAN )15.0 µg/Ml( and CLA )250.0 µg/mL( using mobile phase consisting of 30 mL MeOH: 40 mL 0.2 M OPA at pH 3.0. Flow rate, 1.0 mL/min.

**Fig. S5.** Effect of different % concentration of MeOH on the number of theoretical plates of AMOX )400.0 µg/mL(, VPZ )10.0 µg/mL(, LAN )15.0 µg/mL( and CLA )250.0 µg/mL( using mobile phase consisting of 30 mL ACN: 40 mL 0.2 M OPA at pH 3.0. Flow rate, 1.0 mL/min.

**Fig. S6** Effect of different wavelength on the number of theoretical plates of AMOX) 400.0 µg/mL (, VPZ) 10.0 µg/mL (, LAN) 15.0 µg/mL (and CLA) 250.0 µg/mL) using mobile phase consisting of, consisting of ACN: MeOH: 0.2 M OPA (30: 30: 40) at pH 3.0. Flow rate; 1.0 mL/min.

**Fig. S7** Effect of different flow-rate on the number of theoretical plates of AMOX (400.0 µg/mL), VPZ (10.0 µg/mL), LAN (15.0 µg/mL) and CLA (250.0 µg/mL) using mobile phase consisting of, consisting of ACN: MeOH: 0.2 M OPA (30: 30: 40) at pH 3.0.

**Table S1a Optimization of the chromatographic parameters for separation of the studied mixture (I) by the proposed aqueous liquid chromatographic method.**

| **Resolution (Rs)** | | **No. of theoretical plates(N)** | | | **Parameter** | |
| --- | --- | --- | --- | --- | --- | --- |
| **Rs_2_** | **Rs_1_** | **CLA** | **VPZ** | **AMOX** |  |  |
| *6.85* | *2.1* | *3076* | *2508* | *4455* | **3.0** | **pH** |
| *7.9* | *1.6* | *2029* | *1404* | *2507* | **4.0** |  |
| *7.5* | *1.7* | *1080.4* | *1398* | *1653* | **5.0** |  |
| *5.5* | *1.1* | *1181* | *995* | *1478* | **6.0** |  |
| *6.85* | *2.1* | *3076* | *2508* | *4455* | **210** | **Number of wavelengths** |
| 4.27 | 1.6 | 1755 | 2064 | 3055 | **220** |  |
| 4.68 | 1.79 | 2083 | 1723 | 3085 | **230** |  |
| 4.1 | 1.6 | 1623 | 1705 | 864.9 | **282** |  |
| 7.01 | 2.3 | 1729 | 1438 | 3028 | **25 %** | **Conc. of ACN (%)** |
| *6.85* | *2.1* | *3076* | *2508* | *4455* | **30%** |  |
| 5.3 | 1.79 | 2722 | 2680 | 3985.4 | **35 %** |  |
| 7.54 | 2.4 | 2681 | 1863 | 3069 | **25 %** | **Conc. of MeOH (%)** |
| *6.85* | *2.1* | *3076* | *2508* | *4455* | **30%** |  |
| 3.9 | 1.01 | 2912 | 2118 | 4049.4 | **35 %** |  |
| 7.43 | 1.72 | 2817 | 1690.3 | 3334.1 | **0.8** | **Flow rate (mL/min)** |
| *6.85* | *2.1* | *3076* | *2508* | *4455* | **1.0** |  |
| 5.33 | 1-2 | 1977 | 2604 | 3994.4 | **1.2** |  |

**Table S 1b Optimization of the chromatographic parameters for separation of the studied mixture (II) by the proposed aqueous liquid chromatographic method.**

| **Resolution (Rs)** | | **No. of theoretical plates(N)** | | | **Parameter** | |
| --- | --- | --- | --- | --- | --- | --- |
| **Rs_2_** | **Rs_1_** | **CLA** | **LAN** | **AMOX** |  |  |
| *4.2* | *5.6* | *3376* | *5970* | *4155* | **3.0** | **pH** |
| *3.9* | *5.2* | *1929* | *5404* | *2312* | **4.0** |  |
| *3.1* | *5.4* | *1380.4* | *4995* | *1534* | **5.0** |  |
| *3.08* | *5.8* | *1267* | *3595* | *1356* | **6.0** |  |
| *4.2* | *5.6* | *3376* | *5970* | *4155* | **210** | **Number of wavelengths** |
| 4.17 | 5.05 | 1755 | 4352 | 3055 | **220** |  |
| 4.68 | 4.95 | 2083 | 5023 | 3085 | **230** |  |
| 4.13 | 4.85 | 1623 | 4654 | 864.9 | **282** |  |
| 5.3 | 6.01 | 1897 | 4390 | 3112 | **25 %** | **Conc. of ACN (%)** |
| *4.2* | *5.6* | *3376* | *5970* | *4155* | **30%** |  |
| 4.04 | 4.9 | 2827 | 5874 | 3989 | **35 %** |  |
| 5.1 | 4.8 | 2681 | 4864 | 3069 | **25 %** | **Conc. Of MeOH (%)** |
| *4.2* | *5.6* | *3376* | *5970* | *4155* | **30%** |  |
| 3.3 | 3.69 | 3313 | 5084 | 4156.4 | **35 %** |  |
| 5.3 | 4.72 | 2574 | 5056 | 3542 | **0.8** | **Flow rate (mL/min)** |
| *4.2* | *5.6* | *3376* | *5970* | *4155* | **1.0** |  |
| 3.65 | 4.3 | 2354 | 5698 | 4024.4 | **1.2** |  |

- Where: Number of theoretical plates (N) = 5.45(t_R_/ W_h/2_)^2^ ,
- W_h/2_ is the half peak width
- Resolution (R) = 2 ∆ t_R_/W_1_ +W_2_,
- W_1_and W_2_ are the peaks width of the two components measured at their bases.
- Relative retention (α) = Dm_2_/ Dm_1,_
- Mass distribution ratio (Dm) = t_R_-t_m_/ t_m_

t_R_ is time of retention of the measured substance measured from the injection point and t_m_ is the retention time of a non-retained marker.

**Table S2: robustness data for separation of mixture of two ternary mixtures by the proposed chromatographic method.**

| **%Recovery *** | | | | **Parameter** | |
| --- | --- | --- | --- | --- | --- |
| **CLA** | **LAN** | **VPZ** | **AMOX** |  |  |
| 100.43 | 98.65 | 100.32 | 100.17 | 2.9 | **pH** |
| 99.23 | 99.78 | 100.04 | 99.79 | 3.0 |  |
| 103.568 | 102.44 | 98.45 | 102.39 | 3.1 |  |
| 100.77± 1.91 | 100.19  ± 1.6 | 99.67  ± 0.84 | 100.69  ±1.2 | **$\bar{\text{X}}$± RSD** |  |
| 0.96 | 0.80 | 0.42 | 0.60 | **% Error** |  |
| 100.76 | 101.55 | 98.77 | 99.79 | 0.18 | **Molarity of OPA** |
| 99.23 | 99.78 | 100.04 | 98.3 | 0.2 |  |
| 96.77 | 100.96 | 99.04 | 100.90 | 0.22 |  |
| 99.16  ± 1.73 | 100.54  ± 0.86 | 99.43  ± 0.62 | 99.72  ± 1.08 | **$\bar{\text{X}}$± RSD** |  |
| 0.86 | 0.43 | 0.31 | 0.54 | **% Error** |  |
| 97.99 | 102.6 | 97.99 | 101.87 | 29 % | **Conc. of ACN (%)** |
| 99.23 | 99.78 | 100.04 | 99.79 | 30% |  |
| 101.88 | 98.78 | 98.39 | 100.54 | 31 % |  |
| 99.74  ± 1.63 | 100.26  ± 1.63 | 99.07  ± 1.04 | 100.52  ± 0.96 | **$\bar{\text{X}}$± RSD** |  |
| 0.82 | 0.82 | 0.52 | 0.48 | **% Error** |  |
| 103.12 | 101.77 | 101.67 | 102.32 | 29 % | **Conc. of MeOH (%)** |
| 99.23 | 99.78 | 100.04 | 99.79 | 30% |  |
| 100.95 | 99.87 | 102.08 | 101.76 | 28 % |  |
| 100.79  ± 1.69 | 100.32  ± 0.96 | 100.92  ± 1.11 | 100.94  ± 1.29 | **$\bar{\text{X}}$± RSD** |  |
| 0.85 | 0.48 | 0.56 | 0.64 | **% Error** |  |

*** Each result is the average of three separate determinations**

**Table S3 application of LC method to content uniformity.**

| **Parameter** | **Capsule no.** | **Mixture I**  **Percentage^a^ of the label claim (AMOX)** | **Mixture II**  **Percentage^a^ of the label claim (AMOX)** | **Mixture II**  **Percentage^a^ of the label claim (LAN)** |
| --- | --- | --- | --- | --- |
| **Data** | 1 | 99.96 | 97.98 | 102.07 |
|  | 2 | 98.13 | 99.45 | 101.56 |
|  | 3 | 100.78 | 100.45 | 100.97 |
|  | 4 | 99.08 | 99.32 | 99.45 |
|  | 5 | 100.47 | 98.26 | 100.54 |
|  | 6 | 98.76 | 100.65 | 102.64 |
|  | 7 | 101.56 | 100.85 | 101.32 |
|  | 8 | 100.98 | 101.07 | 99.65 |
|  | 9 | 100.65 | 100.55 | 100.29 |
|  | 10 | 98.43 | 99.83 | 100.84 |
| **Mean** |  | 99.88 | 99.84 | 100.93 |
| **S.D.** |  | 1.20 | 1.08 | 1.01 |
| **% RSD** |  | 1.20 | 1.08 | 1 |
| **% Error** |  | 0.38 | 0.34 | 0.32 |
| **Acceptance value (AV)[3]^b^** | **2.88** | | **2.592** | **2.424** |
| **Max. allowed AV (L1)[3]^b^** | **15.0** | | **15.0** | **15.0** |

1. Each/ result is the average of three separate determinations.
2. USP (2011).

**Table S4 The penalty points for the reported and the proposed methods using Eco-scale:**

| **Parameters** | **Penalty points** | |
| --- | --- | --- |
|  | **Reported method (53)** | **Proposed method** |
| **Reagents and solvents**  **Acetonitrile**  **Methanol**  **O- phosphoric acid**  **n-hexane**  **Triethyl amine** | 4  8  6 | 4  6  4 |
| **Instrument HPLC** | 0 | 0 |
| **Occupational hazard** | 0 | 0 |
| **Waste** | 6 | 5 |
| **Total penalty points** | 24 | 19 |
| **Analytical Eco-score total score** | 76  (acceptable green analysis) | 81  (excellent green analysis) |
